# Potential Therapeutic Use of the Ketogenic Diet in Autism Spectrum Disorders

**DOI:** 10.3389/fped.2014.00069

**Published:** 2014-06-30

**Authors:** Eleonora Napoli, Nadia Dueñas, Cecilia Giulivi

**Affiliations:** ^1^Department of Molecular Biosciences, University of California Davis, Davis, CA, USA; ^2^Medical Investigations of Neurodevelopmental Disorders (M. I. N. D.) Institute, Sacramento, CA, USA

**Keywords:** epilepsy, autism spectrum disorders, dietary intervention, mitochondria, bioenergetics, ketogenic diet, oxidative stress

## Abstract

The ketogenic diet (KGD) has been recognized as an effective treatment for individuals with glucose transporter 1 (GLUT1) and pyruvate dehydrogenase (PDH) deficiencies as well as with epilepsy. More recently, its use has been advocated in a number of neurological disorders prompting a newfound interest in its possible therapeutic use in autism spectrum disorders (ASD). One study and one case report indicated that children with ASD treated with a KGD showed decreased seizure frequencies and exhibited behavioral improvements (i.e., improved learning abilities and social skills). The KGD could benefit individuals with ASD affected with epileptic episodes as well as those with either PDH or mild respiratory chain (RC) complex deficiencies. Given that the mechanism of action of the KGD is not fully understood, caution should be exercised in ASD cases lacking a careful biochemical and metabolic characterization to avoid deleterious side effects or refractory outcomes.

## Biochemistry of the KGD

The ketogenic diet (KGD) is a nutritional approach constituted by high-fat content with adequate protein amount for growth but insufficient levels of carbohydrates for metabolic needs (1), thus forcing the body to primarily use fat as a fuel source. The original KGD was designed as 4:1 lipid:non-lipid (carbohydrate plus protein) ratio with 80% fat, 15% protein, and 5% carbohydrate. Most of the fat is provided as long-chain triglycerides, composing ~80% of the estimated caloric dietary requirement (2). To date, several modifications to the original KGD have been introduced such as lowering the lipid:non-lipid ratio (3) and decreasing the caloric intake from fat (~60–70%) with either no restriction in calorie amount with unlimited protein and fat intake (modified Atkins diet) (4, 5), or with fat provided as triglycerides esterified with medium-chain fatty acids (FA) (to overcome deficits in carnitine metabolism; medium-chain triglyceride diet) (6).

The hormonal changes associated with a KGD include changes in circulating insulin (due to insulin reduction in response to decreasing plasma glucose) and/or leptin (7–9), thus limiting glucose utilization. Under normal conditions, FA mobilized from adipose tissue are catabolized to acetyl coenzyme A (CoA) via β-oxidation, and then oxidized to CO_2_ and H_2_O in the Krebs’ cycle. However, when an imbalance is created between the rate of FA mobilization and the capacity of the Krebs’ cycle to process acetylCoA (e.g., low-carbohydrate and/or protein diet), the liver converts the excess of acetylCoA into ketone bodies (KB), namely acetoacetate (ACA) and d-β-hydroxybutyrate (BHB). A significant fraction of acetone (~30%), the product of the spontaneous decarboxylation of ACA, is found in urine, sweat, and breath (10, 11). KB are utilized as fuel by peripheral tissues sparing glucose and muscle wasting. They generate a comparable amount of energy to protein or carbohydrates (2.7 vs. 4 kcal/g) and, unlike FA, KB can cross the blood–brain barrier (12) constituting the main fuel sources for the brain during fasting periods (13). Most ATP from BHB is via Complex I (70–80%), with the rest via Complex II (14). The low-carbohydrate intake forces the body to sustain systemic glycemia by hepatic gluconeogenesis from non-carbohydrate precursors (e.g., lactate, glucogenic amino acids, and glycerol).

At the center of intermediary metabolism reside mitochondria. These dynamic organelles whose morphology, composition, and function adapt to changes in response to pathological and physiological signals respond to nutritional variations such as those introduced by KGD. Several reports in the literature document changes in mitochondrial number or function in a variety of biological systems, from *in vitro* to *in vivo*, when exposed to KGD or KGD-mimetics (Table 1).

**Table 1:** **Examples extracted from the literature on effects of KGD on mitochondrial function with the potential to benefit ASD symptoms**.

Experimental model	Diet/treatment	KGD-dependent effects	Source
**OUTCOMES RELATED TO ENERGY RESERVES AND/OR ENERGY-SENSING PATHWAYS**			
Rat hippocampus	Young rats fed KGD for 9 weeks	Increased gene expression of mt genes; 46% increase in mitochondria number with no changes in citrate synthase or any other mt enzymatic activity; [PCr]/[Cr] higher (due to lower [Cr])	Bough et al. (15)
Rat hippocampus	Young rats fed KGD for 1 month	Decreased (−30%) body weight than controls; few mt genes overexpressed	Noh et al. (16)
Rat brain	Fed HFD for 3 weeks	[ATP]/[ADP] increased by 12%; lower [Cr] with no changes in [PCr]; lower [cAMP] and [cGMP]	DeVivo et al. (17)
Rat hippocampus	Slices from rat hippocampus (4–7 weeks) with BHB and ACA each at 0.5 or 1 mM	KB prevented rotenone- and 3NP-dependent decrease in ATP and decreased 3NP-dependent ROS production	Kim do et al. (18)
Mouse brain	Mice (8–10 weeks) treated with d-BHB or l-BHB via pumps	BHB restored NADH-supported O_2_ consumption inhibited by MPP^+^, partly the one inhibited by rotenone; BHB increased mtROS. 70–80% ATP from BHB produced via Complex I, the remaining via Complex II	Tieu et al. (19)
Rats	CR-KGD for 7 days	Body weight loss, increased brain expression of IGFR and GLUT3	Cheng et al. (14)
Neuronal human SH–SY5Y cell line	FA (C8 or C10) treatment for 1–6 days	Increased citrate synthase and Complex I activities	Hughes et al. (20)
Rat hippocampus and liver	Rats fed with a 6:1 lipid:non-lipid KGD	Delayed occurrence of epileptic episodes via mTOR inhibition	McDaniel et al. (21)
**OUTCOMES RELATED TO NEUROLOGICAL SYMPTOMS/BEHAVIOR WITH RC COMPLEX AND/OR PDH DEFICIENCIES**			
Child with Leigh syndrome	KGD	Improvement of cerebral lesions by brain MRI	Wijburg et al. (22)
Individuals with PDH deficiency (PDHA1 an PDHX mutations)	KGD (lipid:non-lipid 3:1)	KGD improved only paroxysmal dysfunction	Barnerias et al. (23)
Child, idiopathic PDH deficiency	KGD for ~3 years (lipid:non-lipid 3:1 later switched to 2:1)	Seizure free; improvement in hypotonia, motor development, relationship with environment; poor weight gain, high ketonemia	Di Pisa et al. (24)
Children with PDHE1 mutations	KGD (varied degrees of carbohydrate restriction)	Improved longevity and mental development	Wexler et al. (25)
Child with PHDX	KGD (lipid:non-lipid 4:1, later switched to 3:1 plus MCT oil)	Weight gain, decreased seizure episodes, improved sociability and activity	El-Gharbawy et al. (26)
Children with intractable epilepsy with ETC defects	Age (mean) 45 months, KGD (4:1 lipid:non-lipid) for (mean) 18 months	Eleven of 14 patients decreased seizure frequency by 50–90%; 8 ceased or lowered antiepileptic medications; 8 showed improved cognitive and behavioral functions	Kang et al. (27)
**OUTCOMES RELATED TO MITOCHONDRIAL ANTIOXIDANT DEFENSES AND ROS**			
Mouse hippocampus	Young mice fed a 6:1 lipid:non-lipid KGD for 10–12 d	Decreased mtROS; increases in UCP expression	Sullivan et al. (28)
Rat hippocampus	Adolescent rats, KGD (78% lipid, 0.76% carbs) for 1, 3 days or 1, 3 weeks	KGD-induced initial mild oxidative stress, activation of Nrf2 pathway	Milder et al. (29)
Rat cortex, cerebellum, and hippocampus	Adolescent rats fed with KGD or BHB for 3 weeks	Increased GPX activity and [GSH]	Ziegler et al. (30), Jarrett et al. (31)
Rat neocortical neurons	Neurons exposed to BHB *in vitro*	Decreased Glu-mediated excitotoxicity mtROS production via increased NADH oxidation	Maalouf et al. (32)
**OUTCOMES RELATED TO MITOCHONDRIA-DERIVED NEUROTRANSMITTER METABOLISM**			
Mouse forebrain	Ketotic mice fed KGD (50% lipids) for 3 days	Increased GABA and Gln production	Yudkoff et al. (33)
Cerebrospinal fluid	26 children with refractory epilepsy fed KGD for 6 months	Increased [GABA], [taurine], [Ser], and [Gly]. Higher [GABA] ( >50–90% seizure reduction)	Dahlin et al. (34)
Zebrafish with PDHE1 mutation, lower acetylcholine in inner retina	Larvae fed a mix of lauric/myristic/palmitic acid, and phosphatidyl choline	KGD rescued vision and prolong survival	Maurer et al. (35)
SSDAH mouse model	At PND 12 were fed KGD for 20–30 days	Increased mitochondrial number and size; increased (ATP), no changes in lifespan or neurological outcomes	Nylen et al. (36)

*3-NP, 3-nitropropionic acid; AHA, acetoacetate; BHB, β-hydroxybutyrate; CR-KGD, calorie-restricted ketogenic diet; Cr, creatine; Gln, glutamine; Glu, glutamate; Gly, glycine; GPX, glutathione peroxidase; FA, fatty acids; HFD, high-fat diet; IGFR, insulin-like growth factor receptor; Mt, mitochondrial; MCT, medium-chain triglycerides; Nrf2, Nuclear factor-like 2; PCr, phospho-creatine; PND, post-natal day; Ser, serine*.

## Therapeutic Use of the Ketogenic Diet in Human Diseases

By providing alternative sources of acetylCoA, KGD is the dietary intervention for inborn genetic disorders in pyruvate dehydrogenase (PDH) and glucose transporter 1 (GLUT1) (Table 1), proven effective also in other metabolic conditions, including phosphofructokinase deficiency and glycogenosis type V (McArdle disease) (37). The KGD has also been investigated for the management of neurological disorders such as Alzheimer’s and Parkinson’s diseases (38).

Ketogenic diet has been utilized for >80 years in epilepsy treatment (39, 40) especially in children and adolescents (1, 41) with reduction in seizure frequencies (2, 42) and improvements in developmental progress (26).

Evidence supporting the use of the KGD for patients with intractable epilepsy and respiratory chain (RC) complex defects has been reported in which the majority of patients responded with decreased seizure frequencies, regardless of the RC complex defect or magnitude of deficit (27). The administration of KGD to epileptic patients (37, 39) has been based on the assumption that KB replace glucose as the major metabolic fuel to the brain, although the precise molecular steps still remain obscure. It has been proposed that KB metabolism is not the primary mechanism of this diet, but rather an outcome of the metabolic shifts that occur with this treatment (43) and that the anticonvulsant effects of the KGD could result from an altered gene expression profile accompanied by cellular adaptation mechanisms (15) needed to modify the brain to utilize KB over glucose over time (39).

## Therapeutic Use of KGD in ASD

Autism spectrum disorders (ASD) include a complex neurodevelopmental condition characterized by abnormal social interaction, verbal and non-verbal communication, and limited interest in the surrounding environment associated with stereotyped and repetitive behaviors (44). Limited scientific advances have been made regarding the causes of ASD, with general agreement that both genetic and environmental factors contribute to this disorder (44–47). ASD has been associated to metabolic dysfunction (44, 48) and autism is a common trait of epilepsy-associated diseases (49), and syndromes like Landau–Kleffner, Dravet (50, 51), and Rett (52, 53). Thus, given the beneficial effects of KGD on epilepsy and increased mitochondrial function, its use has the potential to ameliorate some of the ASD-associated symptoms.

Beneficial effects of KGD in children with ASD symptoms have been reported in two independent studies (54, 55). The first study evaluated the role of KGD on 30 ASD children (54). The John Radcliffe diet (a modified medium-chain triglyceride diet with a caloric distribution of 30% in medium-chain triglyceride oil, 30% fresh cream, 11% saturated fat, 19% carbohydrates, and 10% proteins) was administered for 6 months, with intervals of 4 weeks interrupted by two diet-free weeks. Of the 30 children, 40% did not comply or did not tolerate the diet. From the rest, the two children with the milder autistic behaviors showed the most improvement (as judged by total Childhood Autism Rating Scale score, concentration and learning abilities, and social behavior and interactions), while the rest displayed mild to moderate improvements. Interestingly, the beneficial effects of KGD persisted even after termination of the trial. Six of the children enrolled in this study had a higher baseline ketonemia with no apparent PDH and/or RC deficiencies; but it is not clear if any of the other patients underwent this screening, before and/or after the administration of the diet in addition to the lack of the inclusion of a control diet before administering the KGD to the ASD group or during the trial.

The other study (55) reports the administration of a gluten-free casein-free modified KGD (1.5:1 lipid:non-lipid ratio; medium-chain and polyunsaturated FA) for 14-months to a 12-year-old child with ASD and seizures with substantial medical comorbidities associated with a family history of metabolic and immune disturbances. Due to the improvements in seizure activity, improved electroencephalogram, cognitive and social skills, language function, and complete resolution of stereotypies, anticonvulsant medication doses were reduced without worsening of seizures. Of note, the administration of the diet was accompanied by a wealth of medications, a significant weight loss, and transitioning to puberty, so it is difficult to assess the sole role of the diet with this clinical background.

In mouse models of ASD [i.e., Rett syndrome (56), BTBR model (57), and succinate semialdehyde dehydrogenase (SSADH) deficiency (36)], the use of the KGD has improved behavioral abnormalities (increased sociability and decreased self-directed repetitive behavior) and/or decreased the number of seizures, normalized ataxia, and increased lifespan of mutant mice. However, while the KGD was originally designed to be administered under controlled caloric intake (38), most of the mouse studies have been performed under *ad libitum* conditions and/or for a relatively short period [see Ref. (57)]. Moreover, a ketogenic low-carbohydrate diet does not have a significant metabolic advantage over a non-ketogenic low-carbohydrate diet as judged by equal effects in body weight reduction and decreased insulin resistance; however, the former one was associated with higher inflammatory risk and increased perception of fatigue (58).

Although the exact molecular mechanisms underlying the effect of the KGD are still under investigation, several scenarios are reported below to explore the potential therapeutic effects of the KGD in ASD.

### KGD in PDH deficiency

Peripheral blood mononucleated cell (PBMC) from children with high severity scores for ASD has shown impaired PDH activity (44). The KGD is recommended as an alternative source of the acetylCoA in patients (37) with pathogenic mutations in PDH- or GLUT1-encoding genes (22, 25) leading to amelioration of some symptoms (59, 60) especially in those with milder phenotypes (25, 61). Thus, the use of the KGD in ASD with PDH deficiencies might prove to be beneficial.

### KGD in β-oxidation defects

Some patients with ASD have been reported to have defects in fatty acid β-oxidation evidenced as long-chain acyl dehydrogenase deficiency (62) and high concentrations of short or long acyl-carnitines in plasma (63). Carnitine biosynthesis has been recently identified as a risk factor for ASD (64). Thus in these cases, it is advisable to limit the use of a high-fat diet or improve its safety by switching to short or medium-chain FA, which do not utilize the carnitine system.

### KGD in mitochondrial biogenesis

The KGD might improve mitochondrial function by enhancing mitochondrial biogenesis in murine models (15, 65). The medium-chain triglyceride diet (6) has been shown to produce significant increases in citrate synthase and Complex I activity in SH–SY5Y neurons (20). However, the increases in mitochondrial mass would need to result in an OXPHOS outcome of ≥30% [30% as the limit for minor diagnostic criteria of mitochondrial RC disorder (66)] for that particular tissue, given that each tissue has a different ATP threshold (67). Otherwise the increases in mass might not be sufficient to rescue the already impaired ATP production in ASD individuals. Moreover, given the presence of mitochondrial DNA (mtDNA) deletions in PBMC from ASD (44, 68, 69), the KGD-driven mitochondrial biogenesis may result in an enrichment of defective mitochondria due to the proliferating advantage of damaged or deleted mtDNA over wild-type (70, 71). Conversely, treatment of cells containing large-scale mtDNA deletions from a patient with Kearns–Sayre syndrome with KB shifted the heteroplasmy between and within cells (72). The observation that KB can distinguish between normal and respiration-compromised cells suggests that the KB may be useful in treating patients with heteroplasmic mtDNA disorders (72).

### Role of the KGD in RC complex deficits

Children with ASD display an array of mitochondrial dysfunction (MD) of differing severity (44, 73–75). Electron transport chain (ETC) deficiencies have been reported in ASD, primarily in Complex I and IV, but also affecting others such as Complex II, III, and IV (44, 73, 74, 76). The prevalence of seizures (41%) has been observed to be significantly higher in individuals with ASD and MD than in the general ASD population (11%) (74), raising the possibility that epileptic episodes observed in ASD might have a mitochondrial origin. Indeed, epilepsy is a recurrent feature of many inherited “classic” mitochondrial disorders, like myoclonic epilepsy with ragged red fibers, mitochondrial encephalopathy with lactic acidosis, and stroke-like episodes (77), and Leigh syndrome (78). In a small study on children with ETC defects (Table 1), the KGD has been proven to reduce epileptic attacks, with far better prognosis among children with Complex I deficits than Complex IV (27). These results are not surprising given that KGD generates more NADH/FADH_2_ than glucose (2 vs. 5).

### Effect of KGD on energy-sensing pathways alterations

Recently, KGD-fed rats showed increased brain expression of insulin-like growth factor receptor (ILGFR) and neuronal GLUT3 (14). The KGD might have a beneficial effect in some ASD cases considering that IGFR is important for brain health throughout life (79–81), and that IGFR and GLUT3 have both been implicated in ASD (82, 83).

Some energy-sensing molecules and metabolism regulators (including the mammalian target of rapamycin, mTOR) have been recently indicated as possible downstream targets of KGD and may be involved in neuroprotective effects associated to the diet (84). Defects in the mTOR pathway have been linked to ASD (85–87). Failure to inhibit mTOR pathway could lead to MD due to decreased mitophagy (88) resulting in an accumulation of dysfunctional mitochondria as observed in a mouse model of ASD with phosphatase and tensin homolog on chromosome ten (*Pten*) gene haploinsuffciency (89). Indeed, inhibition of mTOR has been linked to a delay in the occurrence of the epileptic episodes (90) and KGD-fed rats showed inhibition of the activation of the mTOR pathway in brain (21), thus representing an appropriate treatment to control seizures while enhancing the clearance of defective/damaged mitochondria.

### Antioxidant and neuroprotective role of the KGD

Ketone bodies (without glucose and at concentrations 10-times higher than physiological ones) inhibit mitochondrial reactive oxygen species (ROS) production in rat neurocortical neurons by increasing NADH oxidation following glutamate (Glu) excitotoxicity (32). It has been suggested that the production of NADPH via oxidation of succinate semialdehyde (SSA) into succinate in the Glu decarboxylase (GAD)/γ-aminobutyric acid (GABA) pathway may buffer the redox changes likely to occur in stressful conditions (91–93). However, other mitochondrial NADPH sources are quantitatively more important than SSADH and fatty acid oxidation produces more mitochondrial ROS than pyruvate oxidation (94).

Thus, the use of KGD could be beneficial in ASD given that higher rates of mitochondrial ROS production and compromised cellular antioxidant status (69, 95, 96) have been reported in peripheral cells from children with ASD (44, 68, 69).

### Effect of the KGD on GABAergic and cholinergic systems disturbances

**Figure 1:**
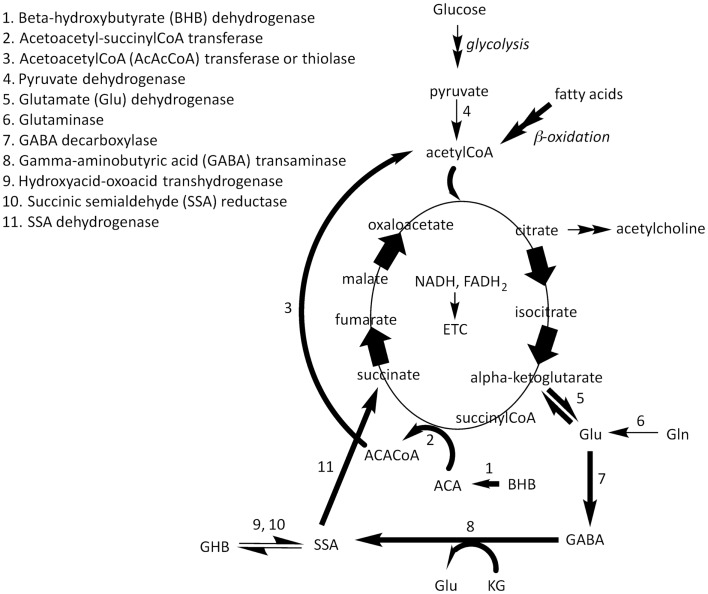
**β-hydroxybutyrate and ACA are utilized as fuel molecules in all mitochondria-containing tissues (except liver)**. BHB is oxidized to ACA by β-hydroxybutyrate dehydrogenase at the inner mitochondrial membrane (arrow 1). ACA acquires the CoA moiety from succinylCoA resulting in succinate and acetoacetylCoA (ACACoA; arrow 2). ACACoA releases acetylCoA catalyzed by ACACoA thiolase (arrow 3). AcetylCoA generated from β-oxidation of fatty acids from the diet and acetylCoA generated by the catabolism of KB is condensed into citrate in the Krebs cycle. The increased flux in the right part of this cycle, increases the concentration of α-ketoglutarate (KG) resulting in increases in the production of Glu via glutamate dehydrogenase (arrow 5) or a transaminase (not shown). Glu from these reactions in addition to that formed from the deamination of glutamine (Gln) via glutaminase (arrow 6) result in the generation of γ-aminobutyric acid (GABA). The GABA shunt bypasses two steps of the Krebs cycle – the KG dehydrogenase complex and the succinyl coenzyme A (CoA) synthase – for the conversion of KG into succinate. It involves three enzymes: a Glu decarboxylase (GAD; arrow 7), which catalyzes the decarboxylation of glutamate to GABA, a GABA transaminase (arrow 8), which converts GABA to succinate semialdehyde (SSA), and an SSA dehydrogenase (arrow 11), which catalyzes the oxidation of SSA to succinate. SSA can be reduced to γ-hydroxybutyric acid (GHB) by an alternative reaction catalyzed by either a hydroxyacid–oxoacid transhydrogenase or SSA reductase (arrows 9, 10).

The GABA shunt bypasses two steps of the tricarboxylic acid cycle – the α-ketoglutarate (KG) dehydrogenase complex and the succinylCoA synthase – for the conversion of KG into succinate (Figure 1). It involves three enzymes: a GAD, catalyzing the Glu decarboxylation to GABA, a GABA transaminase, converting GABA to SSA, and an SSADH, catalyzing the oxidation of SSA to succinate (97). This metabolic route (the GAD/GABA pathway) is conserved from bacteria, through yeast and plants, to vertebrates. In higher eukaryotes, SSA can be reduced to γ-hydroxybutyric acid (GHB) by an alternative reaction catalyzed by a GHB dehydrogenase (98–100). It has been proposed that KGD may limit the availability of oxaloacetate to aspartate aminotransferase, an enzyme involved in brain Glu metabolism, resulting in increased Glu or Gln availability to produce GABA (101). The increased conversion of Glu to GABA would be potentially beneficial in ASD (102–105) (Figure 1).

Changes in GABA neurotransmission by KGD might explain the decrease in seizure frequencies and improved behavior observed in Rett syndrome (106). Studies in patients with ASD strongly suggest a dysfunction in the GABAergic system (107–109). However, changes in other components (including Gly, taurine, and GABA) cannot be excluded (34). In the case of SSADH deficiency (SSADH), the KGD may work through restitution of GABAergic neurotransmission (36), although the use of KGD in SSADHD has been strongly argued until more research is performed to test its potential detrimental effects in humans (110). Conversely, ketotic rodents fed on KGD showed no changes in whole brain (GABA) [between brackets = concentrations; (33, 111)]; however, regional (GABA) changes cannot be ruled out (112), in addition to species-specific differences in the expression of GABA receptors subtypes (113, 114). Considering that cerebrospinal fluid from children treated with KGD showed higher (GABA) (34), it would be of interest to evaluate GABA and amino acid concentrations in different brain areas in animal models of ASD fed KGD.

Dysfunction in the cholinergic system has been observed when PDH deficits are present (115) because a block in this enzyme decreases (citrate), the precursor of acetylcholine via citrate lyase (116). Studies in humans and animal models of ASD suggested that dysfunction of the cholinergic system underlies ASD-related behavioral symptoms (117–119). Trials conducted on ASD individuals have shown beneficial effects of galantamine (an acetylcholinesterase inhibitor) in the management of aberrant behaviors in children and adolescents with ASD (120–122). Treatment of BTBR mice with the acetylcholinesterase inhibitor donepezil hydrochloride improved social preference, social interaction and decreased cognitive rigidity (123). Thus, a KGD has the potential to exhibit beneficial effects in individuals with both ASD and PDH deficiency because the metabolism of KB overcomes the decrease in (citrate) (124) and that of (acetylcholine).

## Potential Side Effects of KGD in ASD

Several side effects of KGD have been reported, among them: (a) limitation in protein, carbohydrate, and other nutrients intake can result in a lack of weight gain and growth inhibition (42), which could be detrimental in ASD because of a predisposition for being underweight (125) and the presence of eating disorders (126). Thiamine, lipoic acid, and l-carnitine supplementation have been helpful in selected cases (25). (b) Dyslipidemia from KGD (127, 128) would need to be supervised in ASD patients with β-oxidation deficits, including carnitine deficiency (64, 129) and, for older patients, the additional increased risk in heart disease and atherosclerosis (130). These patients should limit their fat intake or a modified KGD possibly with carnitine and/or coenzyme Q10 supplementation (131), should be used (132). (c) KGD has an increased risk of systemic ketosis, which may result in lower affinity of hemoglobin for oxygen, resulting in severe outcomes (e.g., coma and death) especially in anemic ASD patients (133). (d) Adverse events experienced by patients with RC complex deficits and epilepsy, which could be extrapolated to those with ASD, included symptomatic persistent hypoglycemia, persistent metabolic acidosis, aspiration pneumonia, and pneumonia followed by respiratory failure (27). (e) Initial fasting and prolonged caloric restriction can cause acute metabolic decompensation in ASD patients with metabolic disorders (134). To reduce the adverse effects of fasting, some studies have omitted the initial fasting period and substituted it with a gradual increase in calories (135). (g) Other side effects include constipation, slower growth, kidney stones, and gastroesophageal reflux (136), although most of them are treatable and/or preventable.

## Concluding Remarks

More research is necessary to understand the potential therapeutic use of KGD in ASD as discussed at length for SSADHD (110). More specifically, how this diet may improve mitochondrial function in ASD and how this putative improvement derived from a better energy and/or neurotransmitter management may influence behavioral symptoms. There are concerns about utilizing KGD in patients with metabolic encephalopathies, with specific contraindications in pyruvate carboxylase deficiency, fatty acid oxidation disorders, and Krebs cycle disorders. Thus, given that the mechanism of action of KGD has not been yet fully understood, even in cases of improved behavioral symptoms, KGD in ASD might need to be prescribed on a case-by-case basis, upon careful biochemical characterization and metabolic profiling.

## Author Contributions

All authors contributed to the design of the work and interpretation of the literature, drafted the work, and gave final approval of the version to be published. All authors agree to be accountable for all aspects of the work in ensuring that questions related to the accuracy or integrity of any part of the work are appropriately investigated and resolved.

## Conflict of Interest Statement

The authors declare that the research was conducted in the absence of any commercial or financial relationships that could be construed as a potential conflict of interest.
